# Identifying the pattern of immune related cells and genes in the peripheral blood of ischemic stroke

**DOI:** 10.1186/s12967-020-02463-0

**Published:** 2020-08-03

**Authors:** Zijian Li, Yueran Cui, Juan Feng, Yanxia Guo

**Affiliations:** Department of Neurology, Shengjing Hospital of China Medical University, No. 36 Sanhao Street, Heping District, Shenyang, 110004 Liaoning People’s Republic of China

**Keywords:** Ischemic stroke, Weighted gene co-expression network analysis, Bioinformatics analysis, Immune cell subtype distribution pattern, Pathological process

## Abstract

**Background:**

Ischemic stroke (IS) is the second leading cause of death worldwide which is a serious hazard to human health. Evidence suggests that the immune system plays a key role in the pathophysiology of IS. However, the precisely immune related mechanisms were still not been systematically understood.

**Methods:**

In this study, we aim to identify the immune related modules and genes that might play vital role in the occurrence and development of IS by using the weighted gene co-expression network analysis (WGCNA). Meanwhile, we applied a kind of deconvolution algorithm to reveal the proportions of 22 subsets of immune cells in the blood samples.

**Results:**

There were total 128 IS patients and 67 healthy control samples in the three Gene Expression Omnibus (GEO) datasets. Under the screening criteria, 1082 DEGs (894 up-regulated and 188 down-regulated) were chosen for further analysis. A total of 11 clinically significant modules were identified, from which immune-related hub modules and hub genes were further explored. Finally, 16 genes were selected as real hub genes for further validation analysis. Furthermore, these CIBERSORT results suggest that detailed analysis of the immune subtype distribution pattern has the potential to enhance clinical prediction and to identify candidates for immunotherapy. More specifically, we identified that neutrophil emerge as a promising target for IS therapies.

**Conclusions:**

In the present study, we investigated the immune related gene expression modules, in which the SLAMF1, IL7R and NCF4 may be novel therapeutic targets to promote functional and histological recovery after ischemic stroke. Furthermore, these hub genes and neutrophils may become important biological targets in the drug screening and drug designing.

## Background

Stroke is one of the leading causes of death and disability in the world, of which ischemic stroke accounts for more than 80%. The burden of ischemic stroke is increasing rapidly with the acceleration of social aging and urbanization, the prevalence of unhealthy lifestyles, the exposure of cardiovascular risk factors [1]. Therefore, the early diagnosis and treatment of IS are faced with great challenges, which need to be further strengthened. During the development of stroke, inflammatory cell infiltration can stimulate intense immune response and cause dysfunction of immune microenvironment in central nervous system, which may further exacerbate the disease [2]. At present, some researches indicate that immunoregulation can delay disease progression as well as improve neurological function and prognosis, which further emphasize the importance of restoring immune microenvironmental homeostasis in the central nervous system [3, 4]. Therefore, in addition to conventional treatment, immunoregulatory therapy as an alternative treatment method is worthy of in-depth study. However, there are limited systematic studies on immune-related gene modules and peripheral blood immune cell subtype distribution patterns in stroke patients.

With the extensive applications and constant development of the gene chip technology, weighted gene co-expression network analysis is increasing used to analysis huge amounts of gene expression data, which is a powerful systematic biological method to analyze the molecular mechanism and network relationship [5]. WGCNA is used to look for co-expressed gene modules, to explore associations between gene networks and interesting sample characteristics, such as health and disease samples, as well as hub genes in the network [6]. In this study, we used the differentially expressed genes between ischemic stroke patients and healthy control in three GEO datasets to construct a co-expression network by WGCNA. We attempted to identify promising immune related candidate biomarkers or potential therapeutic targets of IS from modules in which highly correlated genes clustered. Furthermore, the study of specific molecular and biological functions of these hub genes may better for understanding the underlying mechanisms of IS.

Recent years, a computational analytical tool named CIBERSORT was established to provide an estimation of the abundances of member cell types in a mixed cell population, using gene expression microarray data [7, 8]. By applying CIBERSORT, we assessed the relative proportions of immune cells in 23 blood samples of healthy people and 69 blood samples of ischemic patients from GSE58294 dataset. The obtained immune cell profiles provided proportion and activation status of 22 immune cell subtypes.

In this study, the analysis of the differentially expressed genes between IS patients and healthy people is conducive to revealing the immune related signal pathway mechanism, finding effective targets for the treatment of IS, and laying a foundation for the development of immunoregulatory treatment programs for IS.

## Materials and methods

### Microarray datasets collection

The GEO database (http://www.ncbi.nlm.nih.gov/geo) was used to obtain the gene expression profiles of ischemic stroke. The inclusion criteria were the following: (1) the whole-genome expression profiling of whole blood or peripheral mononuclear cells of IS patients or healthy control samples were available in the datasets; (2) every included dataset contains no less than 20 stroke samples and/or 20 healthy control samples. The exclusion criteria of datasets in this study were set as follows: (1) the IS patients which received any treatment before collection of samples; (2) the control samples were people with definite risk of cardiovascular and cerebrovascular events. The gene expression profiles of stroke GSE58294, GSE22255, GSE16561 and GSE37587 were downloaded. The details of these microarray data were listed in the Table 1.

**Table 1:** Basic information of gene expression profiling

GEO Accession ID	Platform	Samples (Total number)	Number of cases	Number of controls	Country	Year	Author
Training set							
GSE58294	GPL570	Whole blood samples (92)	69 ischemic stroke samples	23 controls	USA	2014	Boryana Stamova
GSE22255	GPL570	Peripheral blood mononuclear cells (40)	20 ischemic stroke samples	20 sex- and age-matched controls	Portugal	2011	Sofia A Oliveira
GSE16561	GPL6883	Whole blood samples (63)	39 ischemic stroke samples	24 healthy controls	USA	2010	Taura L. Barr
Validation set							
GSE37587	GPL6883	Whole blood samples (68)	68 ischemic stroke samples	–	USA	2015	Taura L. Barr

### Data preprocessing and study design

The series matrix of each expression datasets was obtained from GEO database. R software (version 3.5.1) was used to perform the bioinformatics analysis [9]. The “affy” package in R was used to conduct the normalization and background correction of data. Then, the probe level data were then converted into gene expression values. For multiple probes corresponding to a gene, the average expression value was taken as the gene expression value. The difference between batches were eliminated of total 195 samples including 128 stroke patient samples and 67 healthy control samples by ComBat in the “sva” R package. The distribution patterns of disease and control samples (before and after batch correction with ComBat) was observed by principal component analysis (PCA). The flow diagram of this study is shown in Additional file 1: Figure S1.

### Identification of differentially expressed genes in IS

The “limma” package in R was used to obtained differentially Expressed Genes (DEGs) between the stroke patient samples and the healthy control samples in the expressing data. Then we carried out the significance analysis of microarrays and set the select criteria as false discovery rate (FDR) value < 0.05 and Fold change > 1.2 for further network construction.

### Construction of co-expression network

The “WGCNA” package in R was used to construct the co-expression network based on the expression data profile of DEGs. The microarray quality was checked by the “impute” package in R which could detect whether the genes had missing values, and ensure they were good samples. We performed sample clustering to plot the sample tree and detect outliers. Then, we performed Pearson’s correlation matrices for pair-wise genes and found the soft thresholding power β value by using the pickSoftThreshold function of WGCNA.

### Gene Ontology (GO) and Kyoto encyclopedia of genes and genomes (KEGG) analysis

GO Enrichment and KEGG pathway analysis of all genes in hub modules were performed by using the online database STRING (Search Tool for the retrieval of Interacting Genes/Proteins). Furthermore, the PPI network was construct by STRING and Cytoscape 3.7.1.

### Identification of hub genes

First, we defined hub modules as module in which the absolute value of the Pearson’s correlation of module membership > 0.2 and p-value < 0.05. Furthermore, we defined hub genes in co-expression network as genes both satisfies two conditions: the absolute value of the Pearson’s correlation of module membership > 0.8 and the absolute value of the Pearson’s correlation of gene trait relationship > 0.2, which represented high module connectivity and high clinical significance, respectively. Genes in the hub modules were analyzed to construct a protein‐protein interaction (PPI) network, and we defined hub genes in PPI network as genes with a connectivity degree (set default filter as degree in and out) of > 8. Then, we obtained the real hub genes by taking the intersection of hub genes in co-expression network and hub genes in PPI network.

### Evaluation of immune cell subtype distribution

CIBERSORT is a kind of deconvolution algorithm, by which the normalized gene expression matrix can be transformed into the composition of infiltrating immune cells. During the CIBERSORT calculating, the abundance of specific cell types in complex tissue was quantified, and the results of CIBERSORT have been validated by fluorescence activated cell sorting (FACS). LM22 was used as a reference expression signature with 1000 permutations. More accurately forecast of immune cell composition was defined as the CIBERSORT output of p-value < 0.05. Then samples satisfying constraint were selected for further analysis. In this study, the dataset GSE58294 including 69 ischemic stroke samples and 23 healthy control samples were used to estimate the infiltrated immune cells at the time of the stroke. The 22 kinds of infiltrated immune cells included B cells (naïve B cells and memory B cells), T cells (CD8^+^ T cells, naïve CD4^+^ T cells, memory resting CD4^+^ T cells, memory activated CD4^+^ T cells, follicular helper T cells, regulatory T cells and γδ T cells), NK cells (resting NK cells and activated NK cells), monocytes, macrophages (M0 macrophages, M1 macrophages, M2 macrophages), dendritic cells (resting dendritic cells and activated dendritic cells), mast cells (resting mast cells and activated mast cells), eosinophils and neutrophils. All evaluated 22 kinds of immune cell type fractions were to sum up to 1 for each sample.

### Validation of hub genes by microarray dataset

The dataset GSE37587 including 68 stroke samples was used for the validation of the real hub genes. The validation samples were composed by stroke samples of GSE375887 and healthy control samples of GSE16561. We examined the expression of real hub genes in these samples. Differences were considered statistically significant at p-value < 0.05.

### Sample collection and blood routine examination

This study was approved by the ethical committee of Shengjing Hospital, China Medical University (No. 2017PS161K). We obtained informed consent from all participating individuals. The peripheral blood samples were obtained from fifteen patients with ischemic stroke. Fifteen healthy individuals were used as control samples. Clinical characteristics of these samples were listed in Additional file 2: Table S2. Ten milliliters peripheral blood were divided into equal amounts for blood routine examination and quantitative real time polymerase chain reaction, respectively. Blood routine examination was determined by COULTER GEN.S blood cell five-classification analyzer, and blood ingredients (especially neutrophil percentage) were measured by VCS (volume, conductivity, scatter) methods.

### Quantitative real time polymerase chain reaction (qRT-PCR)

Subsequently, qRT-PCR was used to verify the expression of the 16 hub genes in peripheral blood of clinical samples. Total RNA was isolated from each sample using Takara RNAiso Plus (9108) Trizol reagent according to the manufacturer’s instructions. Reverse transcription from total RNA to cDNA and qRT-PCR were performed using the Takara PrimeScript RT Master Mix (RR036A) and SYBR Green Premix (RR420A), respectively. The results were analyzed using the 2−ΔΔCt method and represented as fold changes, normalized to GAPDH. The PCR primers used in this study were shown in Additional file 2: Table S1. Statistically significant was considered as the p-value < 0.05.

## Results

### Data preprocessing and identification of DEGs

**Fig. 1:**
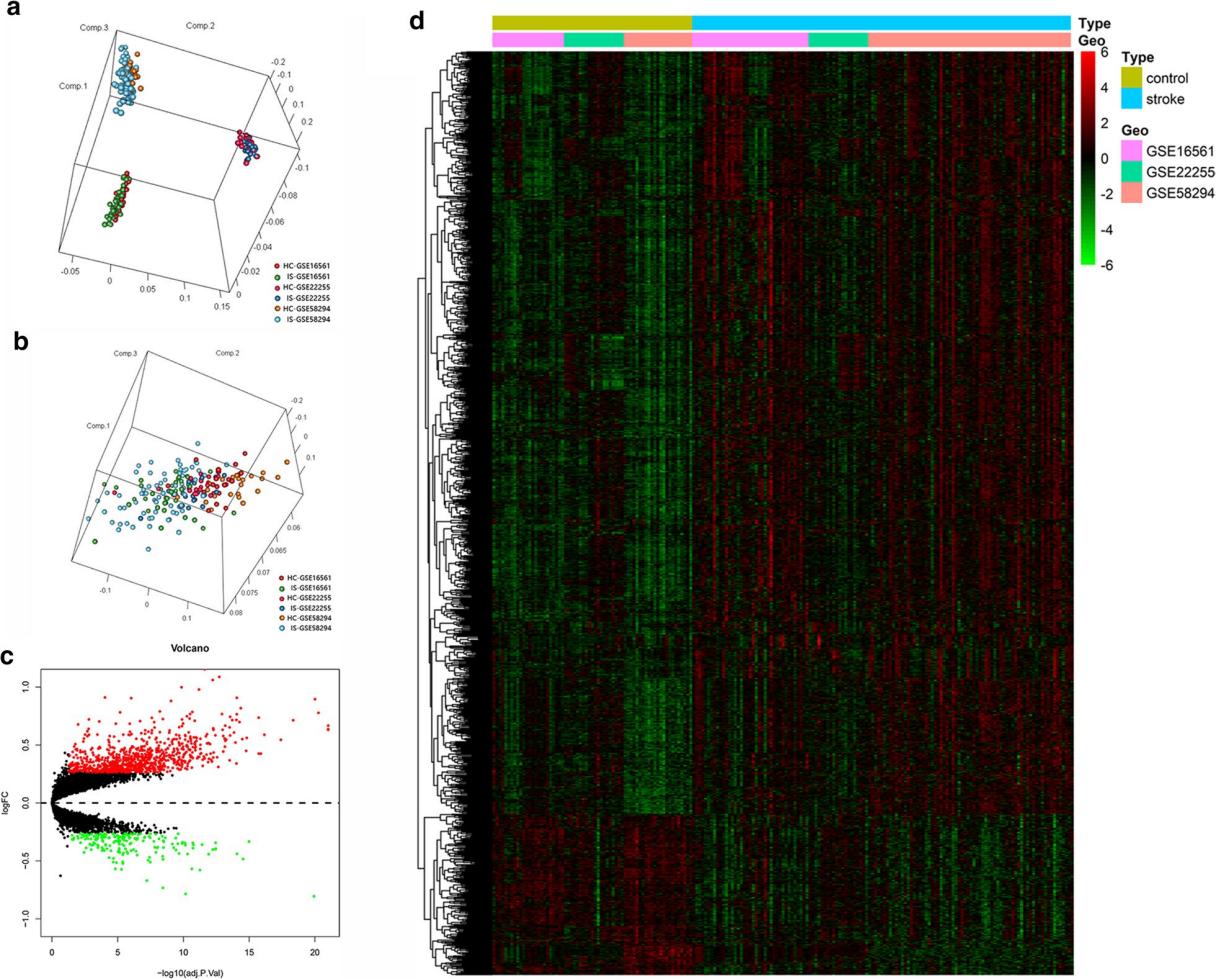
Data Preprocessing and identification of DEGs. **a** PCA for stroke and healthy control samples before batch correction with ComBat. **b** PCA for stroke and healthy control samples after batch correction with ComBat. **c** The volcano plot of DEGs. **d** The heatmap of DEGs

After batch correction with ComBat, the scatter plot of PCA shown that two significantly different distribution patterns between ischemic stroke patient and healthy control samples. Samples of ischemic stroke were mainly distributed on the left side of plot, while healthy control samples were mainly distributed on the right side, as shown in Fig. 1b.

The expression matrixes of three GEO datasets including 128 IS patients and 67 healthy control samples downloaded. Samples of GSE58294, GSE22255 and GSE16561 were merged into one training data set which containing a total of 13,250 genes. After data merge and eliminate the difference between batch, we obtained the expression matrices from the 195 samples in training dataset. Under the threshold of FDR < 0.05 and fold change > 1.2, a total of 1082 DEGs (894 up-regulated and 188 down-regulated) were chosen for further analysis. The volcano plot and heatmap of DEGs were shown in Fig. 1c, d. The expression matrices of total 1082 DEGs in training set were showed in Additional file 3.

### Training set quality assessment and co-expression network construction

As shown in Additional file 4: Figure S2A, seven samples (GSM416554, GSM1406037, GSM1406051, GSM1406046, GSM1406045, GSM1406041, GSM1406055) were removed as outliers from subsequent analysis. Then, we matched the disease state of samples with their expression matrixes. The remaining 188 samples were re-clustered and the sample dendrogram and trait heatmap were plotted (Additional file 4: Figure S2B). In this study, the soft-thresholding powers was chosen as β = 8 where the curve first reached R^2^ = 0.79, to construct a weighted network based on a scale-free topology criterion (Additional file 5: Figure S3A–D).

### Identification of clinically significant modules

**Fig. 2:**
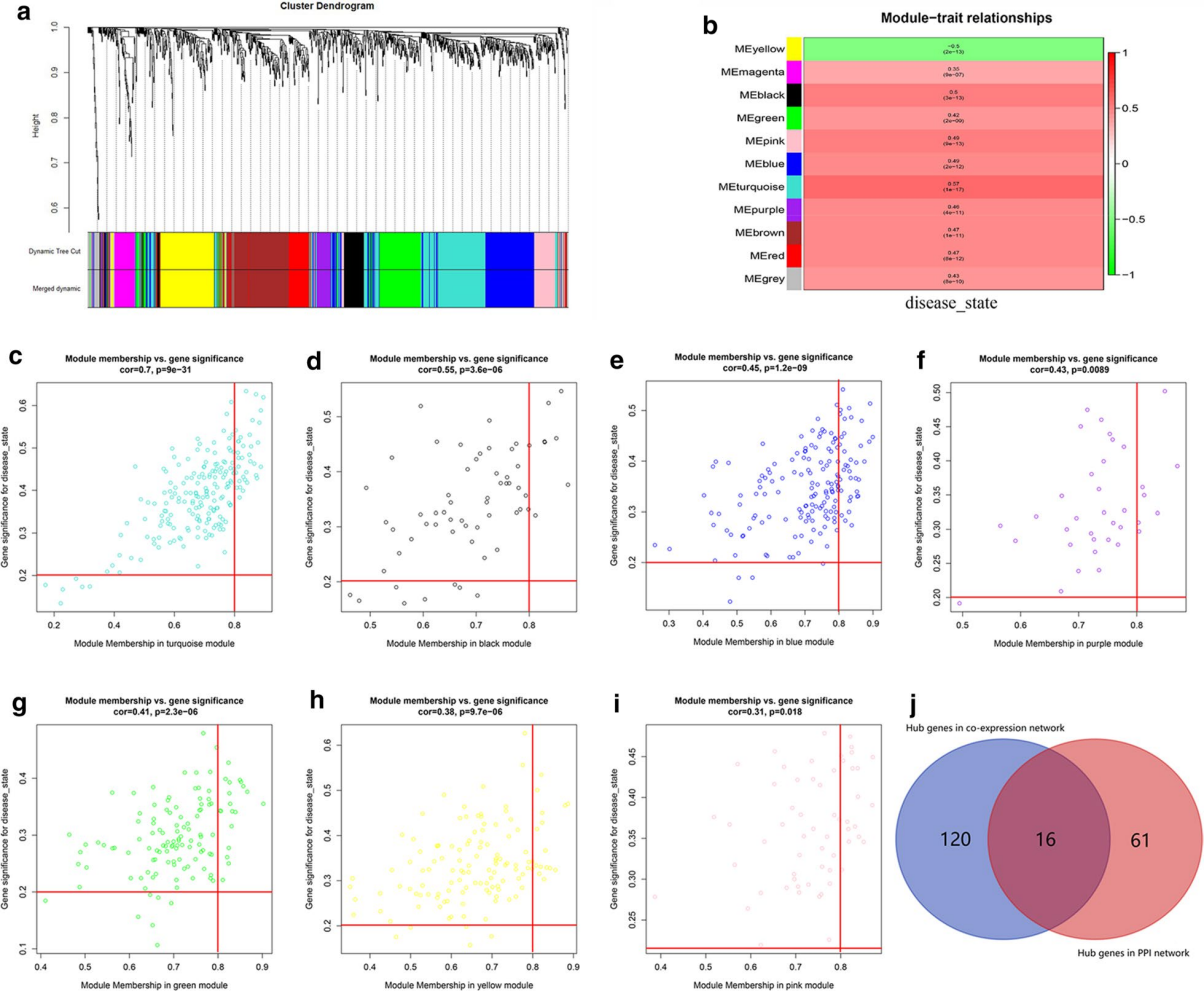
Cluster dendrogram and module-trait relationship plot. **a** Dendrogram of all differentially expressed genes clustered based on a dissimilarity measure (1-TOM). **b** Heatmap of the correlation between module eigengenes and ischemic stroke. Scatter diagrams for module membership vs. gene significance of disease state. **c** Turquoise module. **d** Black module. **e** Blue module. **f** Purple module. **g** Green module. **h** Yellow module. **i** Pink module. **j** Venn plot of hub genes in the co-expression and PPI networks

A total of 11 modules were identified through the dynamic tree cutting method, as shown in Fig. 2a. And the number of genes in each module was listed in Table 2. After relating modules to traits, high correlations were observed in the trait of disease state (healthy control or ischemic stroke), as shown in Fig. 2b. Clinically significant modules were considered as the p-value < 0.05 and eligible for further analysis, which included turquoise module, black module, blue module, purple module, green module, yellow module and pink module.

**Table 2:** The number of genes in the 11 modules

Module colors	Number
Black	62
Blue	166
Brown	152
Green	124
Grey	43
Magenta	47
Pink	58
Purple	36
Red	66
Turquoise	200
Yellow	128

### Identification of hub genes

136 hub genes were selected from the clinically significant modules in the co-expression network by WGCNA **(**Fig. 2c–i and Additional file 6: Figure S5), and 77 genes were selected through PPI network analysis (Additional file 7: Figure S4A-D and Additional file 8: Figure S6). As listed in Table 3, 16 hub genes in both the co-expression and PPI networks were selected as real hub genes for further validation analysis (Fig. 2j).

**Table 3:** The real hub genes in the co-expression and PPI networks

GeneSymbol	ModuleColor	GS.disease state	p.GS. disease state	MM.of related module	p.MM.of related module
TLR8	Turquoise	0.435661339	4.14E−10	0.871990002	1.33E−59
PTEN	Turquoise	0.428731523	8.34E−10	0.874600844	2.22E−60
IFNAR1	Turquoise	0.407160397	6.68E−09	0.818666737	1.12E−46
CCR7	Yellow	− 0.41442059	3.37E−09	0.810615379	4.24E−45
IL7R	Yellow	− 0.31537227	1.04E−05	0.816302419	3.31E−46
SLAMF1	Yellow	-0.333846288	2.84E−06	0.808174198	1.23E−44
TIMP2	Blue	0.447586714	1.20E−10	0.899305925	1.00E−68
CANT1	Blue	0.390141685	3.12E−08	0.82537424	4.68E−48
PAK1	Blue	0.501249842	2.36E−13	0.804602912	5.74E−44
ATG7	Blue	0.432671718	5.61E−10	0.802242306	1.56E−43
NCF4	Blue	0.293300473	4.41E−05	0.807809113	1.45E−44
ITGAM	Blue	0.541718688	9.94E−16	0.811555078	2.80E−45
WAS	Blue	0.479687326	3.29E−12	0.866722618	4.37E−58
MYD88	Blue	0.447728134	1.18E−10	0.80773727	1.49E−44
MAPK1	Blue	0.430201337	7.20E−10	0.864080663	2.39E−57
FGR	Blue	0.402714638	1.01E−08	0.834484479	5.05E−50

*TLR* toll like receptor 8, *PTEN* phosphatase and tensin homolog, *IFNAR1* interferon alpha and beta receptor subunit 1, *CCR7* C–C motif chemokine receptor 7, *IL7R* interleukin 7 receptor, *SLAMF1* signaling lymphocytic activation molecule family member 1, *TIMP2* tissue inhibitor of metalloproteinases-2, *CANT1* calcium activated nucleotidase 1, *PAK1* p21 (RAC1) activated kinase 1, *ATG7* autophagy related 7, *NCF4* neutrophil cytosolic factor 4, *ITGAM* integrin subunit alpha M, *WAS* WASP actin nucleation promoting factor, *MYD88* MYD88 innate immune signal transduction adaptor, *MAPK1* mitogen-activated protein kinase 1, FGR proto-oncogene, Src family tyrosine kinase, FGR

### Functional enrichment annotation

To further explore the function of each module, we performed the GO and KEGG pathway analysis. The GO analysis results showed that genes in hub modules were mainly enriched in immune response, immune system process, immune effector process, neutrophil degranulation and leukocyte activation (terms shared by three or more modules). The detailed top-ten GO function annotation terms of each module were selected and listed in the Table 4 (according to p-value). The KEGG pathways of each module were listed in Table 5, which mainly enriched in pathways of Complement and coagulation cascades, NOD-like receptor signaling pathway, Th17 cell differentiation, Th1 and Th2 cell differentiation and T cell receptor signaling pathway and so on. Most of these pathways were associated with immune and inflammatory responses.

**Table 4:** The top 10 GO enrichment terms of genes in each module

GO term ID	Term description	Observed gene count	Background gene count	False discovery rate
Black module				
GO:0002376	Immune system process	29	2370	4.39E−08
GO:0006955	Immune response	24	1560	4.39E−08
GO:0002237	Response to molecule of bacterial origin	12	317	3.03E−07
GO:0071345	Cellular response to cytokine stimulus	18	953	3.5E−07
GO:0032496	Response to lipopolysaccharide	11	298	1.13E−06
GO:0019221	Cytokine-mediated signaling pathway	14	655	5.05E−06
GO:0009617	Response to bacterium	13	555	5.46E−06
GO:0010033	Response to organic substance	27	2815	7.47E−06
GO:0007166	Cell surface receptor signaling pathway	23	2198	2.47E−05
GO:0071310	Cellular response to organic substance	23	2219	2.67E−05
Blue module				
GO:0043312	Neutrophil degranulation	45	485	4.58E−29
GO:0002366	Leukocyte activation involved in immune response	47	616	5.58E−28
GO:0045055	Regulated exocytosis	48	691	4.66E−27
GO:0006887	Exocytosis	50	774	5.35E−27
GO:0002443	Leukocyte mediated immunity	46	632	1.14E−26
GO:0006955	Immune response	65	1560	6.26E−26
GO:0002252	Immune effector process	52	927	1.49E−25
GO:0045321	Leukocyte activation	50	894	2.16E−24
GO:0001775	Cell activation	51	1024	8.55E−23
GO:0016192	Vesicle-mediated transport	63	1699	2.08E−22
Green module				
GO:0002252	Immune effector process	19	927	0.0071
GO:0002274	Myeloid leukocyte activation	15	574	0.0071
GO:0002443	Leukocyte mediated immunity	16	632	0.0071
GO:0001775	Cell activation	19	1024	0.009
GO:0002366	Leukocyte activation involved in immune response	14	616	0.009
GO:0002697	Regulation of immune effector process	11	362	0.009
GO:0043312	Neutrophil degranulation	13	485	0.009
GO:0045321	Leukocyte activation	18	894	0.009
GO:0006810	Transport	44	4130	0.0163
GO:0006887	Exocytosis	15	774	0.0163
Pink module				
GO:0051345	Positive regulation of hydrolase activity	12	742	0.0037
GO:0001932	Regulation of protein phosphorylation	12	1370	0.021
GO:0001933	Negative regulation of protein phosphorylation	8	422	0.021
GO:0002252	Immune effector process	10	927	0.021
GO:0002275	Myeloid cell activation involved in immune response	9	519	0.021
GO:0002376	Immune system process	18	2370	0.021
GO:0002444	Myeloid leukocyte mediated immunity	9	519	0.021
GO:0006334	Nucleosome assembly	5	137	0.021
GO:0006810	Transport	24	4130	0.021
GO:0006955	Immune response	15	1560	0.021
Purple module				
GO:0043312	Neutrophil degranulation	11	485	8.96E−07
GO:0006952	Defense response	14	1234	1.28E−06
GO:0002376	Immune system process	18	2370	2.07E−06
GO:0001819	Positive regulation of cytokine production	9	390	2.31E−06
GO:0006955	Immune response	15	1560	2.31E−06
GO:0045321	Leukocyte activation	12	894	2.33E−06
GO:0032940	Secretion by cell	12	959	4.74E−06
GO:0016192	Vesicle-mediated transport	15	1699	5.49E−06
GO:0006950	Response to stress	18	3267	0.00015
GO:0009056	Catabolic process	13	1859	0.00059
Turquoise module				
GO:0006955	Immune response	58	1560	1.55E−14
GO:0002376	Immune system process	71	2370	3.57E−14
GO:0002252	Immune effector process	42	927	8.96E−13
GO:0045087	Innate immune response	36	676	1E−12
GO:0006952	Defense response	46	1234	1.89E−11
GO:0050896	Response to stimulus	128	7824	4.85E−09
GO:0006950	Response to stress	73	3267	9.15E−09
GO:0080134	Regulation of response to stress	41	1299	6.2E−08
GO:0034097	Response to cytokine	36	1035	7.1E−08
GO:0048584	Positive regulation of response to stimulus	53	2054	8.11E−08
Yellow module				
GO:0002684	Positive regulation of immune system process	23	882	4.91E−05
GO:0002250	Adaptive immune response	13	280	8.36E−05
GO:0008284	Positive regulation of cell population proliferation	22	878	8.36E−05
GO:0007166	Cell surface receptor signaling pathway	36	2198	0.00011
GO:0050851	Antigen receptor-mediated signaling pathway	9	122	0.00011
GO:0048584	Positive regulation of response to stimulus	34	2054	0.00014
GO:0048583	Regulation of response to stimulus	50	3882	0.00019
GO:0002376	Immune system process	36	2370	0.00031
GO:0006955	Immune response	28	1560	0.00031
GO:0046649	Lymphocyte activation	13	358	0.00031

**Table 5:** The KEGG pathway enrichment analysis of genes in each module

KEGG term ID	Term description	FDR
Black module		
hsa04668	TNF signaling pathway	0.0029
hsa04610	Complement and coagulation cascades	0.0065
hsa04621	NOD-like receptor signaling pathway	0.0068
Blue module (top 10)		
hsa04142	Lysosome	0.0015
hsa04670	Leukocyte transendothelial migration	0.0015
hsa04380	Osteoclast differentiation	0.0078
hsa05152	Tuberculosis	0.0078
hsa00052	Galactose metabolism	0.0079
hsa05140	Leishmaniasis	0.0136
hsa04140	Autophagy—animal	0.0233
hsa04666	Fc gamma R-mediated phagocytosis	0.0286
hsa05161	Hepatitis B	0.032
hsa05322	Systemic lupus erythematosus	0.032
Green module		
hsa04610	Complement and coagulation cascades	0.0028
hsa05133	Pertussis	0.0132
hsa04010	MAPK signaling pathway	0.0394
Pink module		
hsa05322	Systemic lupus erythematosus	0.0161
Purple module		
hsa04145	Phagosome	0.00051
hsa00380	Tryptophan metabolism	0.0024
hsa00340	Histidine metabolism	0.0176
hsa04066	HIF-1 signaling pathway	0.0176
hsa04640	Hematopoietic cell lineage	0.0176
hsa04216	Ferroptosis	0.0327
hsa00480	Glutathione metabolism	0.0396
hsa04979	Cholesterol metabolism	0.0396
Turquoise module (top 10)		
hsa05167	Kaposi’s sarcoma-associated herpesvirus infection	2.06E−05
hsa05161	Hepatitis B	4.52E−05
hsa05162	Measles	0.00013
hsa05164	Influenza A	0.00013
hsa05321	Inflammatory bowel disease (IBD)	0.00024
hsa05140	Leishmaniasis	0.00042
hsa04620	Toll-like receptor signaling pathway	0.00045
hsa04012	ErbB signaling pathway	0.00088
hsa04660	T cell receptor signaling pathway	0.0019
hsa04917	Prolactin signaling pathway	0.0019
Yellow module		
hsa05340	Primary immunodeficiency	4.86E−05
hsa04060	Cytokine-cytokine receptor interaction	0.00079
hsa04662	B cell receptor signaling pathway	0.00079
hsa04640	Hematopoietic cell lineage	0.0018
hsa04650	Natural killer cell mediated cytotoxicity	0.006
hsa04659	Th17 cell differentiation	0.0143
hsa04660	T cell receptor signaling pathway	0.0143
hsa04514	Cell adhesion molecules (CAMs)	0.0455
hsa04658	Th1 and Th2 cell differentiation	0.0493

The results of the GO and KEGG pathway analysis confirmed that modules listed below, especially blue module, turquoise module and yellow module may play key roles in the immune system, such as immune response, immune cell activation and differentiation.

### Verification of hub genes using the dataset

**Fig. 3:**
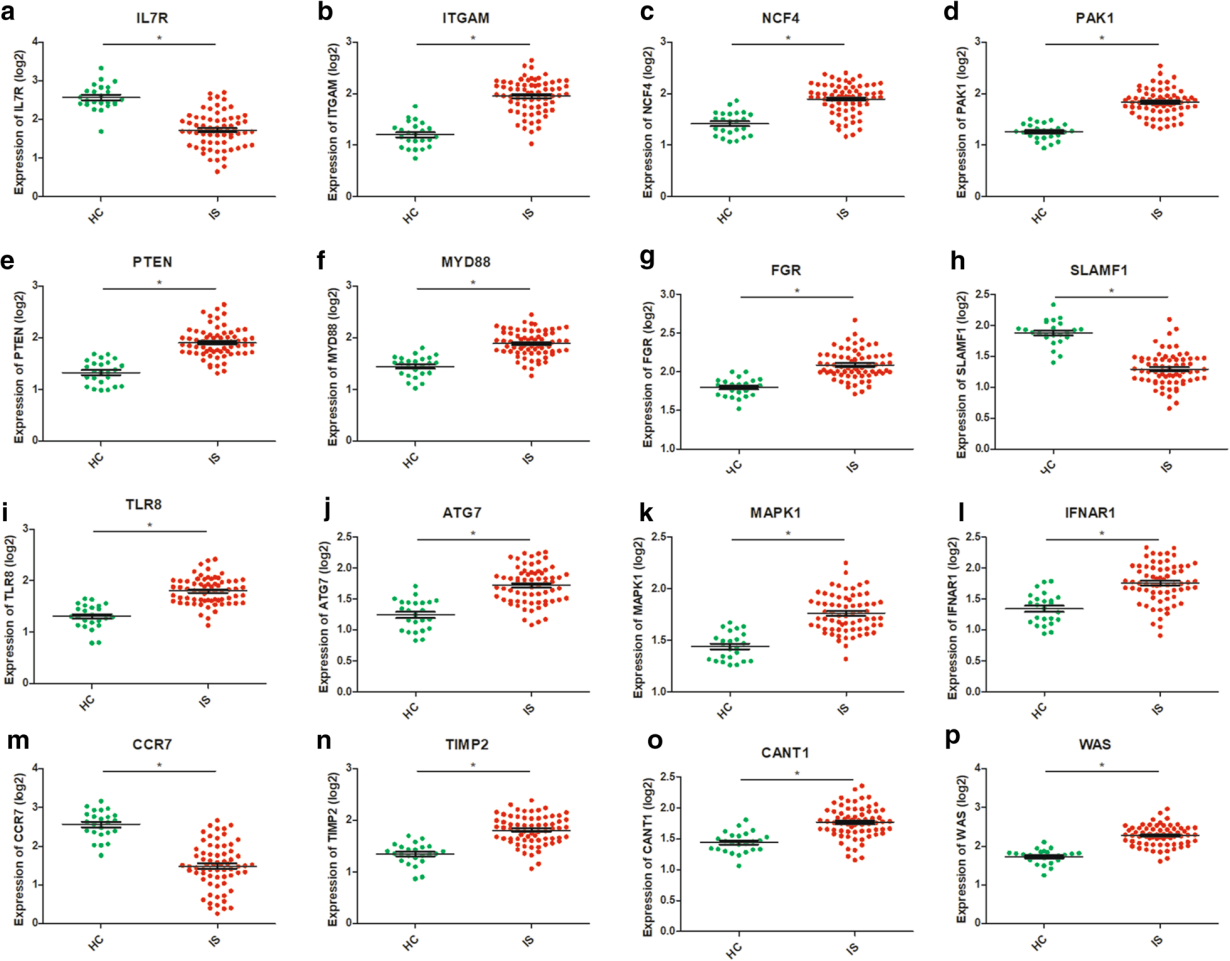
Verification using GSE37587 dataset. Green: healthy control samples; red: ischemic stroke samples. Statistically significant was considered as the p-value < 0.05. **a**–**p** Verification of the expression of IL-7R, ITGAM, NCF4, PAK1, PTEN, MYD88, FGR, SLAMF1,TLR8, ATG7, MAPK1, IFNAR1, CCR7, TIMP2, CANT1 and WAS, respectively, in the HC and IS group of validation cohort

The validation cohort was made up of stroke patient samples in GSE37587 and healthy control samples in GSE16561. The two data sets used the same chip on the same platform, making the data merge reasonable. After the difference between batches were eliminated, standardized data was used for the validation of 16 hub genes. As shown in Fig. 3a–p, the validation results of total 16 genes were consistent with the multiple chips conjoint analysis. We found ITGAM, NCF4, PAK1, PTEN, MYD88, FGR, TLR8, ATG7, MAPK1, IFNAR1, TIMP2, CANT1 and WAS were upregulated in IS samples and IL7R, SLAMF1 and CCR7 were downregulated in IS samples.

### Profile of immune cell subtype distribution pattern

**Fig. 4:**
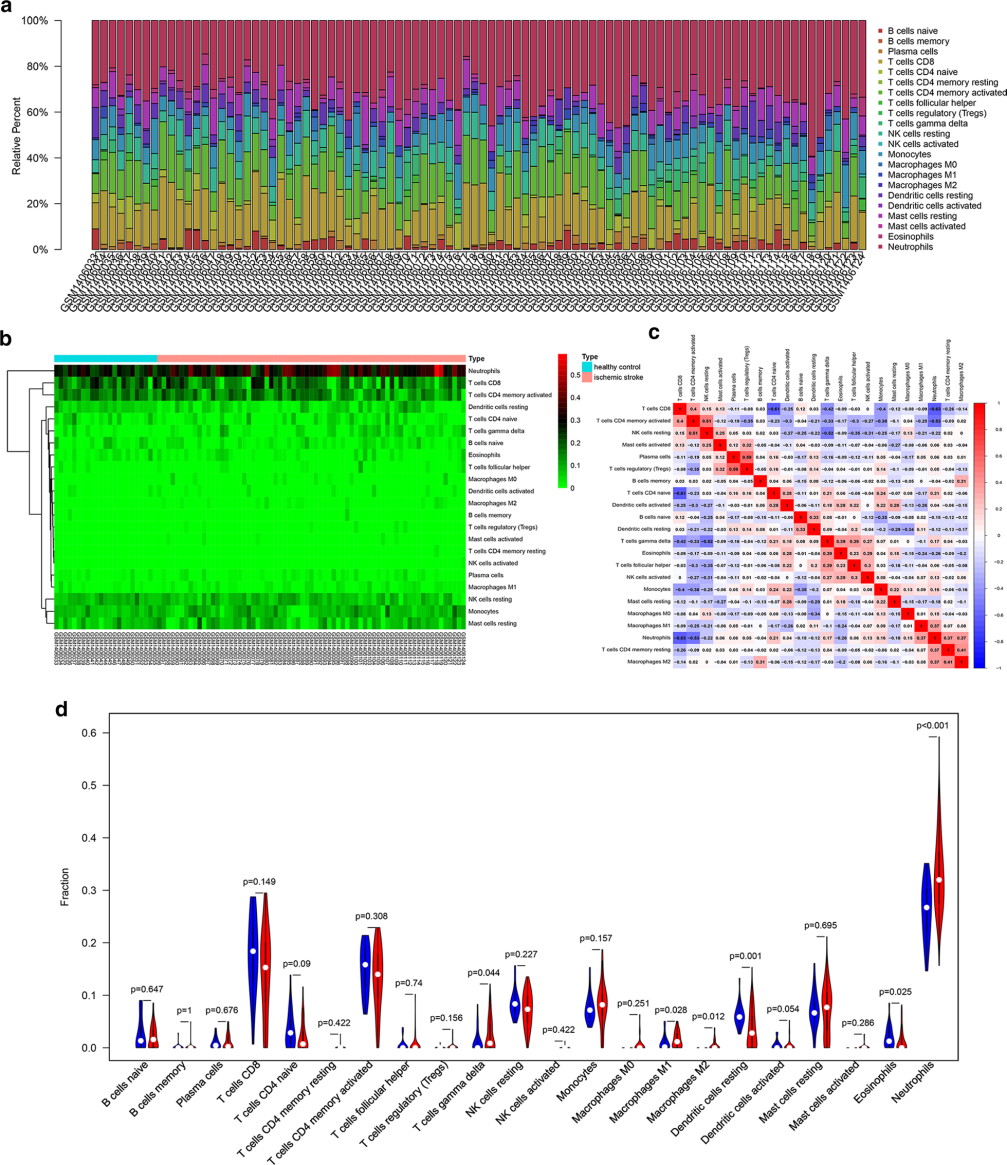
The profiles of immune cell subtype distribution pattern in GSE58294 cohort. **a** The bar plot visualizing the relative percent of 22 immune cell in each sample. **b** Heatmap of the 22 immune cell proportions in each sample. **c** Correlation heatmap of all 22 immune cells. **d** Violin plot of all 22 immune cells differentially infiltrated fraction

As shown in Fig. 4a, the histogram shows the general distribution of various immune cells in each sample. Different colors represent different types of immune cells. The height of each color represents the percentage of such cells in the sample, and the sum of the percentage of various immune cells is 1. It was observed that neutrophils, resting mast cells, M1 and M2 macrophages, monocytes, CD8^+^ T cells, activated memory CD4^+^ T cells, γδ T cells and naïve B cells are the main infiltrating cells. The distribution of some immune cell subsets with low abundance expression in stroke has not been fully revealed, due to the limitations of CIBERSORT algorithm. Proportions of immune cells from two comparison group samples showed individual differences, and the cluster analysis of infiltrated immune cells in disease and normal data is an important means for discovering pathological processes and immunoregulatory mechanisms (Fig. 4b). As shown in Fig. 4c, the proportions of different infiltrated immune cell subpopulations were weakly to moderately correlated. For instance, the correlation of neutrophils and CD8^+^ T cells is 0.63, and the correlation of CD8^+^ T cells and naïve CD4^+^ T cells is 0.61 and so on. Compared with normal samples, stroke samples generally contained a higher proportion for γδ T cells (p = 0.044), macrophages M1 (p = 0.028), macrophages M2 (p = 0.012) and neutrophils (p < 0.001), whereas the resting dendritic cells (p = 0.001) and eosinophils (p = 0.025) fraction was relatively lower (Fig. 4d).

**Fig. 5:**
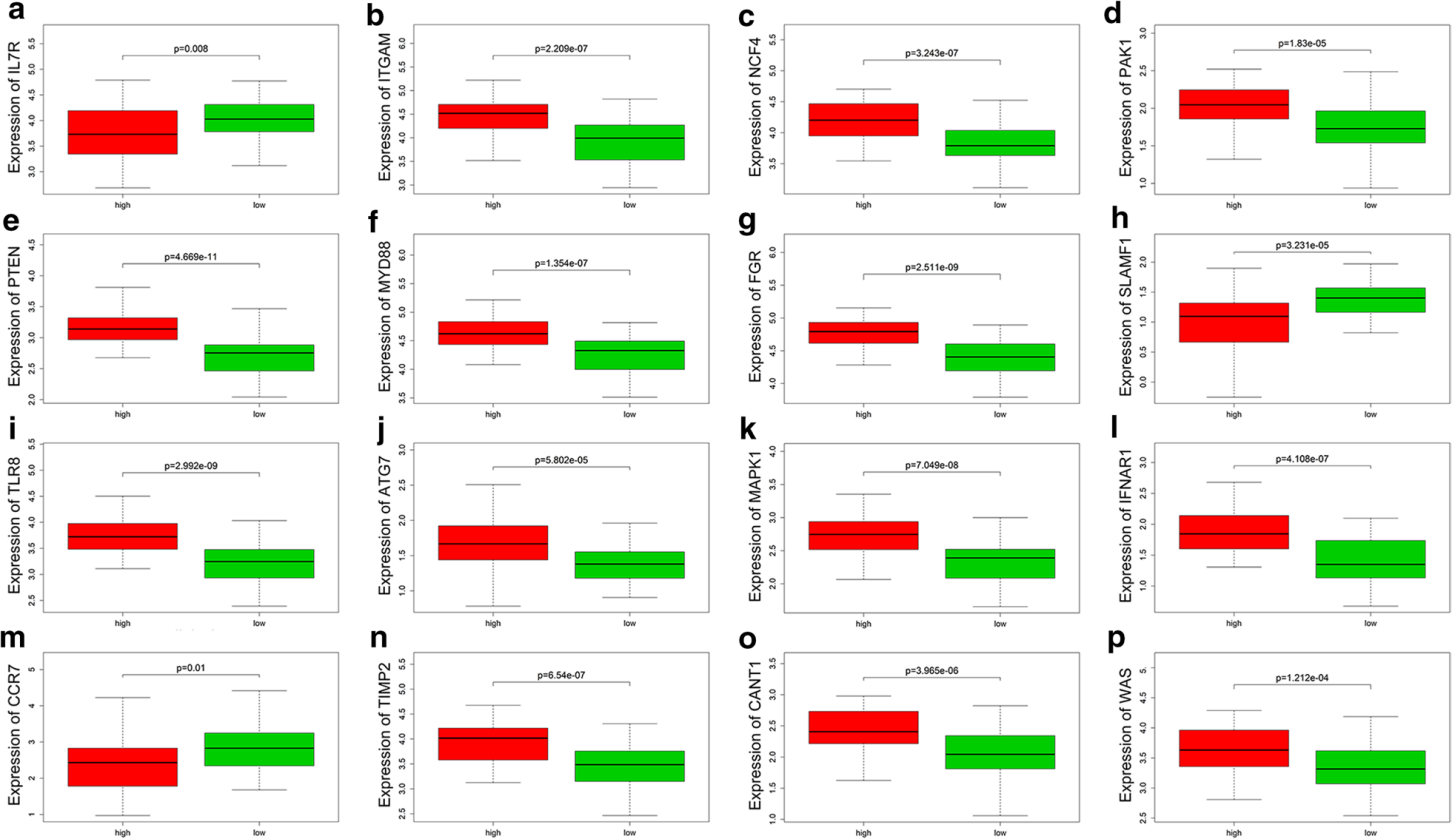
Correlation analysis of hub genes expression and the proportion of neutrophils. Red bar: samples with a high proportion of neutrophils; green bar: samples with a low proportion of neutrophils. Statistically significant was considered as the p-value < 0.05. **a**–**p** Correlation analysis of the proportion of neutrophils and expression of IL-7R, ITGAM, NCF4, PAK1, PTEN, MYD88, FGR, SLAMF1, TLR8, ATG7, MAPK1, IFNAR1, CCR7, TIMP2, CANT1 and WAS, respectively

For further analysis the correlation between hub genes expression and proportions of neutrophils infiltrating, we divided the sample into high proportion neutrophils infiltrated group and low proportion neutrophils infiltrated group. The results of Wilcoxon test indicated that the expression of hub genes were significantly correlated with proportions of neutrophils infiltrating (TLR8, p = 2.99E−09; MAPK1, p = 7.05E−08; PAK1, p = 1.83E−05; PTEN, p = 4.67E−11; IFNAR1, p = 4.11E−07; CCR7, p = 0.010322089; SLAMF1, p = 3.23E−05; IL7R, p = 0.00816272; TIMP2, p = 6.54E−07; MYD88, p = 1.35E−07; CANT1, p = 3.96E−06; ATG7, p = 5.80E−05; NCF4, p = 3.24E−07; ITGAM, p = 2.21E−07; WAS, p = 0.000121197; FGR, p = 2.51E−09), as shown in Fig. 5a–p. The proportion of neutrophil distributed in the peripheral blood of stroke patients increased, in which the gene expression of IL7R, SLAMF1 and CCR7 were low, and vice versa. The results are consistent with our studies shown in Fig. 3a–p and Fig. 4d.

### Verification in the clinical samples

**Fig. 6:**
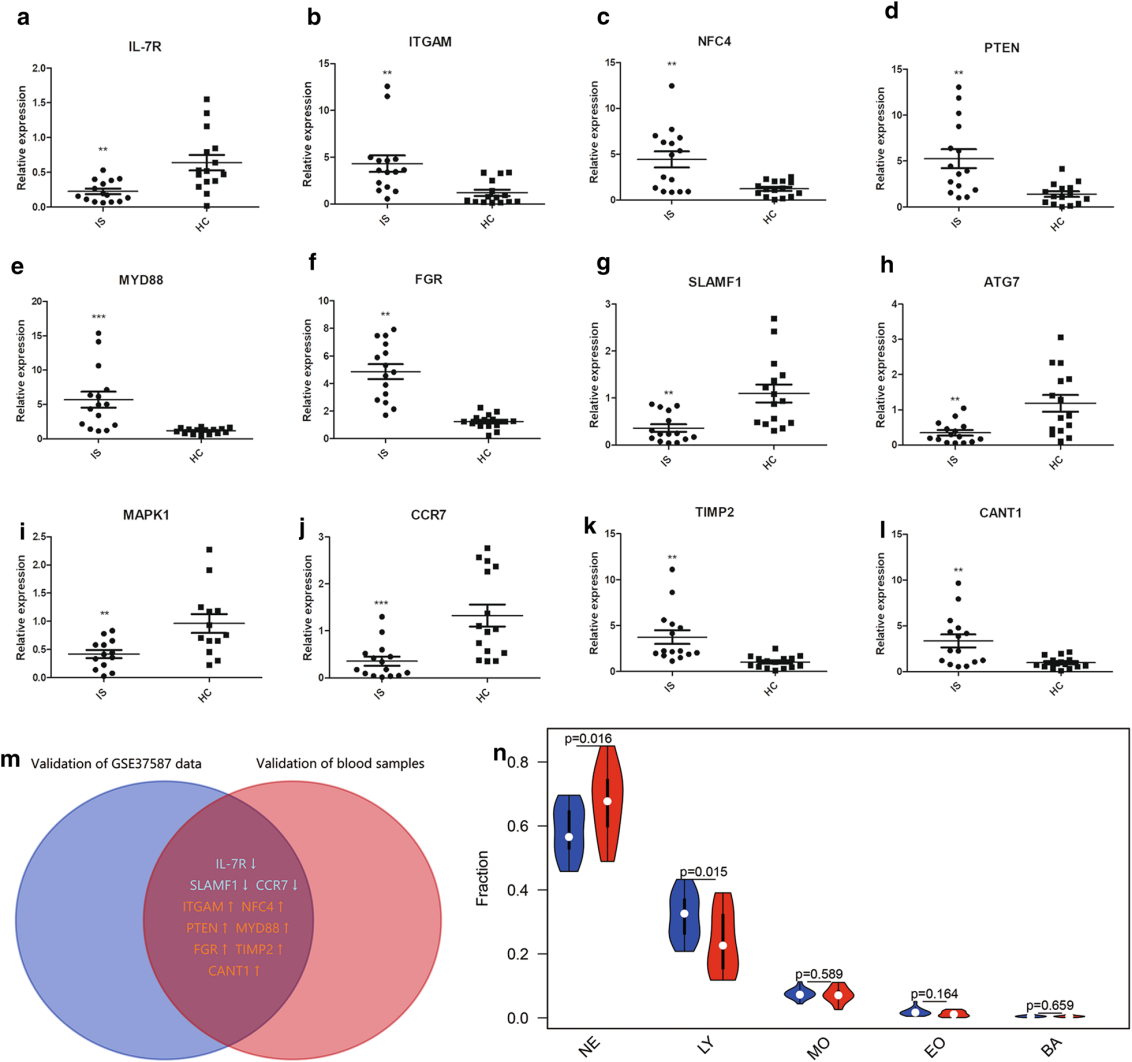
Verification in the clinical samples. **a**–**l** Verification using qRT-PCR analysis. **m** Venn plot of validated hub genes. Red: upregulated genes; blue: downregulated genes. **n** Neutrophils (NE), lymphocytes (LY), monocytes (MO), eosinophils (EO) and basophils (BA) percentage in the clinical samples. *p < 0.05, **p < 0.01, ***p < 0.001 compared to the control group

To further validate the results of the microarray analysis, we examined the expression of the 16 real hub genes which were dysregulated in IS by the qRT-PCR with the blood samples from fifteen IS patients and another fifteen healthy control individuals. And we found that there were 12 genes dysregulated in validation blood samples. Unpaired t test with Welch’s correction was used to make statistics of the data shown in Fig. 6a–l. Finally, these genes in both the GSE37587 dataset and our clinical blood samples were overlapped (Fig. 6m), among these ITGAM, NFC4, PTEN, MYD88, FGR, TIMP2 and CANT1 were upregulated in IS samples and IL-7R, SLAMF1 and CCR7 were downregulated in IS samples. Taken together, despite four hub genes did not show significant, our experiment results were generally consistent with the trends of bioinformatics analysis results. And the results of blood routine examination indicated that the percent of neutrophil granulocyte was increased in acute ischemic stroke patients **(**Fig. 6n). The expansion of harmful neutrophils subsets associated with disease severity may play an important role by promoting systemic inflammation and disruption of the blood–brain barrier.

## Discussion

Ischemic stroke is a severe disease with high incidence and fatality rate and one of the main causes of adult lifetime disability, which leave heavy pressure and burden to patients’ families and society. Therefore, researchers have realized that it is very urgent to improve the early preclinical diagnosis and treatment level. In this study, WGCNA and CIBERSORT algorithms were used to explore the pathological process and marker genes in peripheral blood samples of ischemic stroke. After data preprocessing and weighted gene network construction, the modules were associated with feature and function enrichment analysis. GO and KEGG analysis identified black, blue, green, pink, purple, turquoise and yellow module as immune-related modules. According to the module recognition heatmap and scatter diagrams, the modules related to stroke onset were black, blue, green, pink, purple, turquoise and yellow module (p < 0.05) (Fig. 2b–i). By taking overlap, the 7 hub modules are obtained for further analysis. Finally, we found out 16 hub genes distributed across 3 modules. There are TLR8, PTEN and IFNAR1 in turquoise module, CCR7, IL7R and SLAMF1 in yellow module, TIMP2, CANT1, PAK1, ATG7, NCF4, ITGAM, WAS, MYD88, MAPK1 and FGR in blue module.

Tumor suppressor PTEN is highly expressed in neurons. Ischemic stroke induced neuronal PTEN degradation and led to cognitive impairment [10]. However, PTEN has been reported to be inhibited by many microRNA (miRNA) and performed neuroprotective effect against ischemic stroke in experimental models. miR-103 can target PTEN and downregulate its expression, which depletion restrains the progression of atherosclerosis through blocking PTEN-mediated MAPK signaling [11]. Another data demonstrates that miR-130a prevented cerebral ischemia/reperfusion damage against ischemic stroke by mediating the PTEN/PI3K/AKT pathway [12]. On the other side, long non-coding RNA (lncRNA) SETD5-AS1 participates in the development of ischemic stroke by activating PTEN and inhibiting PI3K/AKT pathway [13].

Innate immunity plays an important role in brain ischemic injury, toll-like receptors are innate immunity receptors that activate inflammation and adaptive immunity. Many studies have focused on the role of TLR2 and TLR4 in brain damage caused by ischemic stroke. However, the role of TLR8 in ischemic stroke is relatively rare. Firstly, increasing TLR8 expression was found associated with greater inflammatory response, bigger infarct volumes and poor outcomes [14]. Then, Tang and colleagues found that TLR8 could promote neuronal apoptosis and T cell-mediated inflammation following stroke [15]. Recently, it was reported that TLR8 gene rs3764880 polymorphism might be associated with susceptibility and involved in the inflammatory response and lipid metabolism of ischemic stroke in southern Chinese Han population [16].

Type 1 IFN-stimulated genes reaction in the microglia exposed to ischemia/reperfusion depends on innate immune receptors including TLR4 and IFNAR1 [17], which played a deleterious role in the outcome after stroke [18]. Microglia-targeted IFNAR1 knockdown demonstrated that interferon signaling specifically in microglia is essential for the protection effect of ischemic preconditioning [19]. Furthermore, the IFNAR1 knockdown cells were protected against oxygen–glucose–deprivation induced neuro-inflammation with reduced pro-apoptotic cleaved caspase-3 levels, compared to negative control cells [20].

Our results indicated that there is a negative correlation between the expression of genes in the yellow module and the occurrence of ischemic stroke. Chemokine receptor CCR7 comes from a wide range of sources in the body, including peripheral blood neutrophils, macrophages and glial cells of CNS. Ischemic stroke plays a bipolar role in the peripheral immune response. Peripheral leukocyte activation occurs early, followed by severe immunosuppression, characterized by splenic atrophy and reduced expression of cytokines and chemokines [21]. Peripheral immune cells such as T cells migrate to the brain and exacerbate the developing infarct [22]. Studies have shown that the relative mRNA expression of CCR7 in peripheral blood of patients with traumatic brain injury within 24 h after onset was significantly reduced, compared with the normal control group [23], which was consistent with our experimental results.

Costimulatory molecule and microbial sensor SLAMF1 (also known as CD150) initiates signal transduction pathways in a variety of immune cells [24]. SLAMF1 is overexpressed on the T cell, B cell and their subgroups of Systemic lupus erythematosus (SLE) patients [25, 26]. Expression of SLAMF1 was significantly increased in peripheral blood CD4+, T helper 17, and CD19+ B cells from autoimmune thyroid disease patients [27]. In addition, compared with the healthy control group, SLAMF1 was significantly down-regulated in patients with acute myocardial infarction (AMI) [28]. Evidence has been accumulating that SLAM family members are potential targets for inflammatory and autoimmune diseases [29]. Bacille Calmette-Guérin (BCG) infection significantly upregulated SLAMF1, which enhanced inflammatory response by activating the NF-κB signaling pathway [30]. Yurchenko and colleagues reported that SLAMF1 is a new target for modulation of TLR4-TRAM-TRIF inflammatory signaling in human cells [24]. However, the dysfunction of SLAMF1 in ischemic stroke has not been reported before. In this study, we found that SLAMF1 was downregulated in the IS patient samples which indicate that SLAMF1 may be a potential target for medical intervention in patients with ischemic stroke. And its role in immune regulation needs to be further elucidated.

IL7R widely express in immunity system and play critical roles in immune cell development and immune system homeostasis. Studies showed that the IL7R allele polymorphism was associated with an increased risk of multiple sclerosis (MS) [31], and there was a significant association between the IL7R promoter polymorphisms and the age of onset of MS [32]. Furthermore, the latest evidence shows that the lymphoid-associated interleukin 7 receptor (IL7R) regulates tissue-resident macrophage development [33]. However, the association between IL7R expression and IS onset has not been reported before. In the present study, it was found that IL7R was low expression in IS cohorts. Larger-scale studies of populations and in-depth researches are needed to explore the roles played by the IL7R during the pathogenesis of IS.

Our results indicate that the expression of TIMP-2 and MAPK1 upregulated in IS, which are consistent with the finding of Liu NN and colleagues [34]. In addition, their research illustrates that the TIMP2-dependent MAPK pathway could be negatively regulated by miR-410, which exert neuroprotective role and against oxidative stress-induced apoptosis after IS in mice models. On the other hand, another study indicated that overexpressing TIMP2 and TIMP1 in rat middle cerebral artery occlusion (MCAO) model 3 days before occlusion could receive the larger beneficial effect such as smaller infarct size, and better motor function recovery than autologous bone marrow cells alone or in combination with drugs [35].

CANT1 was reported been associated with chondrodysplasia [36], clear cell renal cell carcinoma [37], with no evidence for an association with IS.

Mitsios and colleagues reported for the first time an upregulated expression of PAK1 in human after IS and rat brain samples following MCAO [38]. PAK1, as a major cyclin-dependent kinase 5 (Cdk5) substrate and target, could be hyperphosphorylated by p35/Cdk5, leading to the downregulation and activity inhibition of PAK1 [39]. PAK1 involved in many actin dynamics pathways, and its expression dysregulation might cause loss of synapses, cognitive deficits, and impaired motor functions.

Lee and colleagues performed genome-wide association studies (GWAS) for small-vessel occlusion (SVO) stroke and the results showed that three single nucleotide polymorphisms in ATG7 were associated with SVO stroke [40]. Cerebral endothelial ATG7 was reported that could modulate pro-inflammatory cytokines expression and lead to brain ischemia/reperfusion injury during stroke [41]. Furthermore, the previous study of our research team [42] has been reported that KCNQ1OT1 promotes autophagy by regulating miR-200a/FOXO3/ATG7 pathway in cerebral ischemic stroke.

NADPH-complex component p40-phox subunit (encoded by NCF4), a key factor in biochemical pathways and the innate immune response. Due to its important role in the innate immune response, the gene polymorphism of NCF4 has been reported to be involved in chronic granulomatous disease [43, 44] and increased the risk of colorectal cancer [45]. Some researches demonstrated that NCF4 might be a potential diagnostic biomarker or a regulatory target for acute myocardial infraction (AMI) [46] and coronary artery disease [47]. We suspect that NCF4 act on similar modes and approaches in cerebral vascular diseases compared to those of the cardiovascular diseases.

ITGAM (also known as CD11b) is predominantly expressed in monocytes, macrophages and granulocytes, involved in various adhesive interactions and mediating the uptake of complement-coated particles. ITGAM genetic variations was reported be associated with susceptibility to SLE [48]. As far as we know, during ischemic stroke CD11b was strongly expressed on the activated macrophage/microglia and infiltrating leukocytes, reflecting the clinical severity of inflammatory response in the brain [49].

To the best of our knowledge, TLR4/MyD88/MAPK/NF-κB signal pathway was one of the classical pathways in ischemic stroke‑induced inflammation. Many drugs [50, 51] and treatments [52] could inhibit the TLR4-mediated inflammatory responses and decrease proinflammatory cytokine release through the MyD88-dependent signaling pathway. MyD88-dependent signaling contributes to the inflammatory responses induced by cerebral ischemia/reperfusion [53].

Some studies indicate that the inflammatory properties of circulating neutrophils increase during acute ischemic stroke [49], which similar to our research results (Fig. 4a). The expansion of harmful neutrophils subsets associated with disease severity may play an important role by promoting systemic inflammation and disruption of the blood–brain barrier. We speculated that these mRNA signal of the analyzed genes comes mainly form neutrophils (related not only to neutrophils number increased, but also to the neutrophils activated). New therapeutic approaches of stroke by rebalancing the neutrophil subset homeostasis may become potential targeted therapies. In addition, modulating macrophages/microglia [54], or Th17/γδ T cells [55] subsets with biologics after stroke have become a topic of interest in recent years, and the discovery of new drugs related to T cell subsets or macrophages/microglia polarization may enable the realization of stroke targeted therapies.

## Conclusion

In conclusion, our study is the first to integrate most comprehensive microarray samples of ischemic stroke for WGCNA. In this study, we demonstrated the 7 immune related gene expression modules and 16 hub genes, in which the SLAMF1, IL7R and NCF4 may be novel candidate biomarkers or therapeutic targets and never reported be related to IS before. Our findings may provide valuable reference for further pathogenesis mechanism elucidation of IS. Furthermore, these hub genes and neutrophils may become important biological targets in the drug screening and drug designing.

## Supplementary information

**Additional file 1: Figure S1.** A workflow of the analysis procedure. HC, healthy control; IS, ischemic stroke; GO, gene oncology; KEGG, Kyoto encyclopedia of genes and genomes.

**Additional file 2: Table S1.** Sequences of primers used for qPCR. **Table S2.** Clinical characteristics of validation samples.

**Additional file 3.** The expression matrices of total 1082 DEGs in training set.

**Additional file 4: Figure S2.** Samples clustering and identification of differentially expressed genes (DEGs) in IS samples. (A) Samples clustering of total 195 samples to detect outliers. (B) Re-clustering of 188 samples: sample dendrogram and trait heatmap. The clustering was based on the expression data of DEGs between healthy controls (HC) and ischemic stroke (IS) patients. In disease state, white means HC and red means IS.

**Additional file 5: Figure S3.** Determination of soft-thresholding power in the WGCNA. (A) Analysis of the scale-free index for a set of soft-thresholding powers (β). (B) Analysis of the mean connectivity for a set of soft-thresholding powers. (C) Histogram of connectivity distribution when β = 8. (D) Checking the scale free topology when β = 8.

**Additional file 6: Figure S5.** Scatter diagrams for module membership vs. gene significance of disease state in brown(A), magenta(B), red(C) and grey(D) module.

**Additional file 7: Figure S4.** PPI network in different modules. (A) Yellow module. (B) Black module. (C) Blue module. (D) Turquoise module. The yellow nodes represent the hub genes (connectivity degree > 8) in each module.

**Additional file 8: Figure S6.** PPI networks of purple(A), pink(B) and green(C) module.

## Data Availability

The datasets during and/or analyzed during the current study available from the corresponding author on reasonable request.

## Abbreviations

IS: Ischemic stroke
WGCNA: Weighted gene co-expression network analysis
GEO: Gene Expression Omnibus
GO: Gene oncology
KEGG: Kyoto encyclopedia of genes and genomes
STRING: Search Tool for the retrieval of Interacting Genes/Proteins
DEGs: Differentially expressed genes
PPI: Protein–protein interaction
miRNA: microRNA
lncRNA: Long non-coding RNA
SLE: Systemic lupus erythematosus
MS: Multiple sclerosis
MCAO: Middle cerebral artery occlusion
GWAS: Genome-wide association studies
AMI: Acute myocardial infraction
