# Supplementary figures and images for: Identifying the pattern of immune related cells and genes in the peripheral blood of ischemic stroke
Source: J Transl Med. 2020 Aug 3;18:296. doi: 10.1186/s12967-020-02463-0 (PMC7398186; doi:10.1186/s12967-020-02463-0)

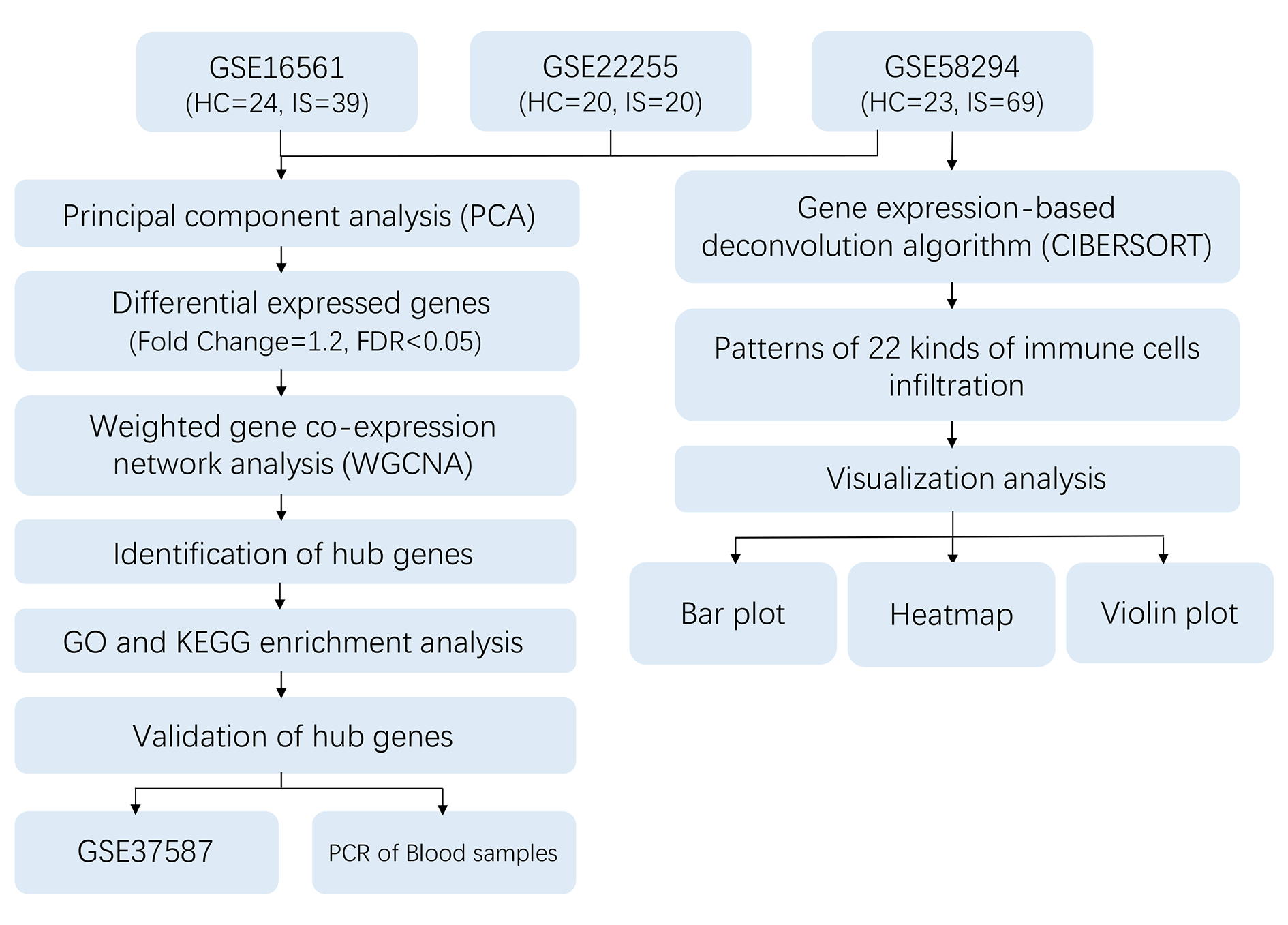

Supplement: Supplementary file 1 — Additional file 1: Figure S1. A workflow of the analysis procedure. HC, healthy control; IS, ischemic stroke; GO, gene oncology; KEGG, Kyoto encyclopedia of genes and genomes. [file 12967_2020_2463_MOESM1_ESM.tif]

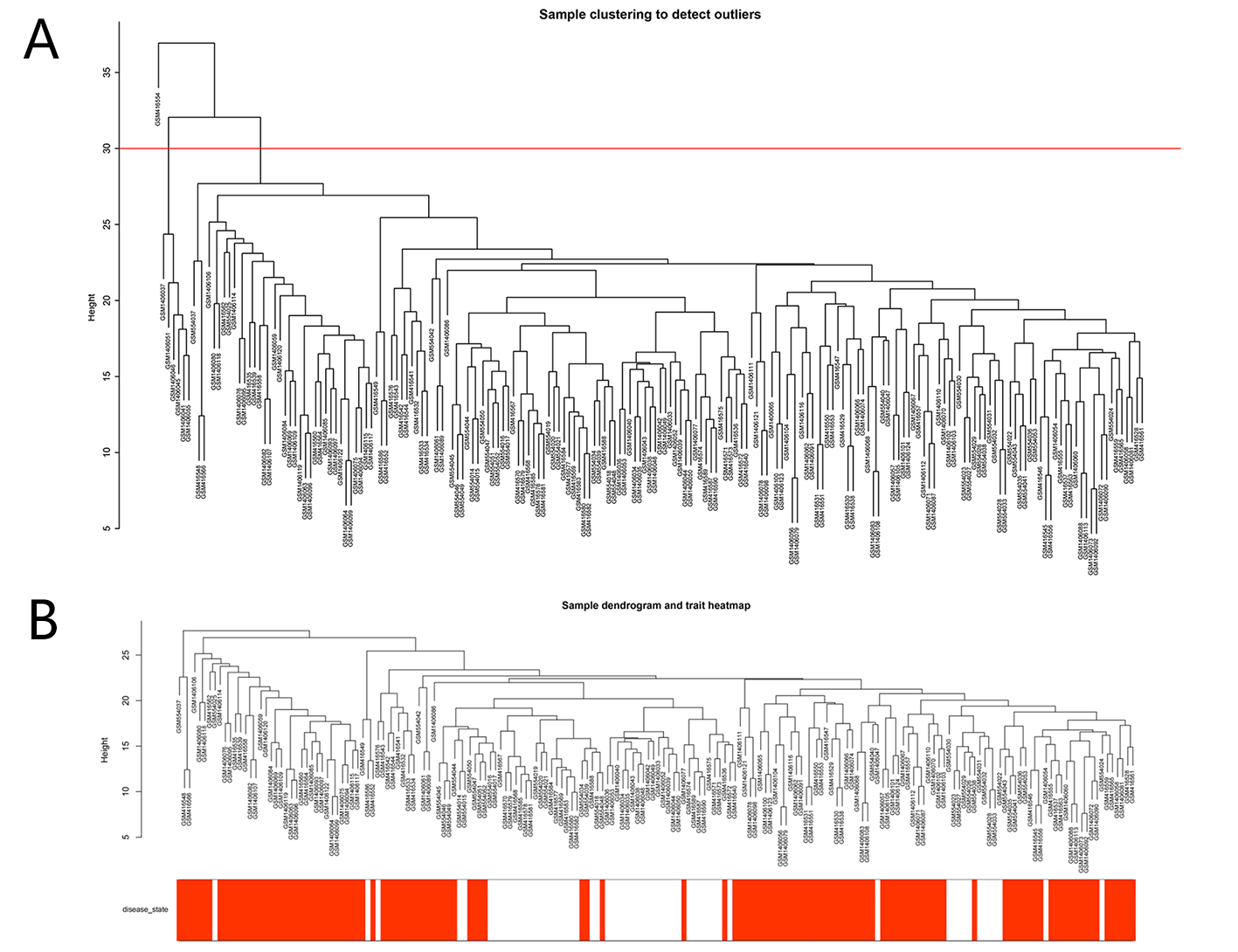

Supplement: Supplementary file 4 — Additional file 4: Figure S2. Samples clustering and identification of differentially expressed genes (DEGs) in IS samples. (A) Samples clustering of total 195 samples to detect outliers. (B) Re-clustering of 188 samples: sample dendrogram and trait heatmap. The clustering was based on the expression data of DEGs between healthy controls (HC) and ischemic stroke (IS) patients. In disease state, white means HC and red means IS. [file 12967_2020_2463_MOESM4_ESM.tif]

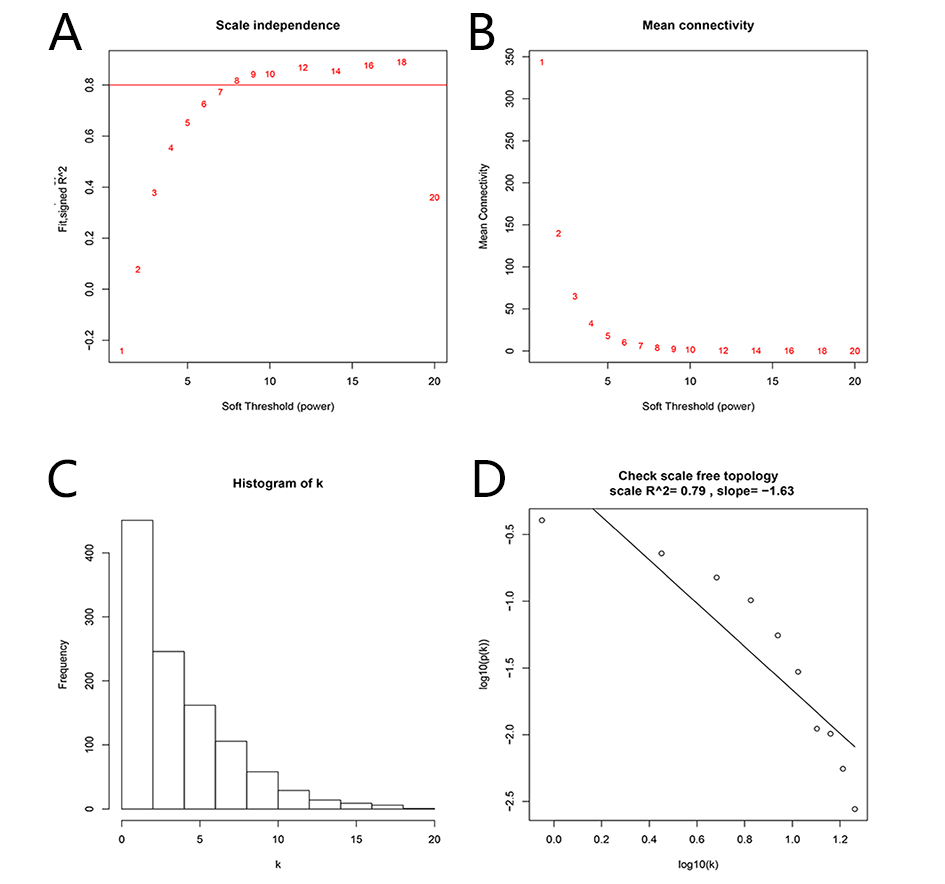

Supplement: Supplementary file 5 — Additional file 5: Figure S3. Determination of soft-thresholding power in the WGCNA. (A) Analysis of the scale-free index for a set of soft-thresholding powers (β). (B) Analysis of the mean connectivity for a set of soft-thresholding powers. (C) Histogram of connectivity distribution when β = 8. (D) Checking the scale free topology when β = 8. [file 12967_2020_2463_MOESM5_ESM.tif]

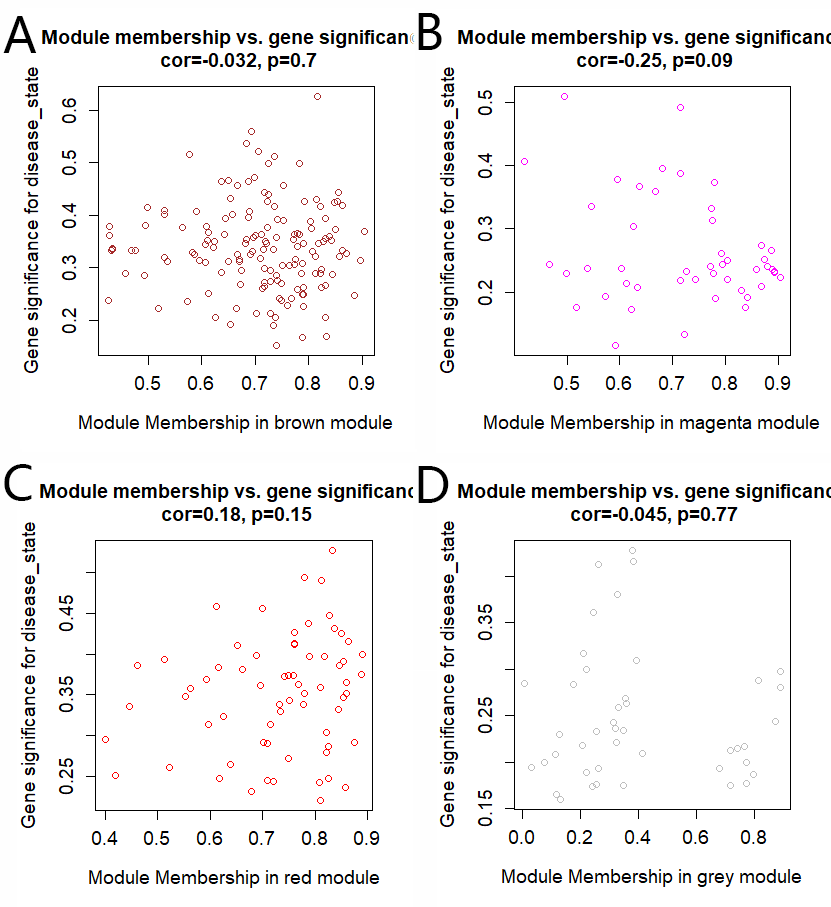

Supplement: Supplementary file 6 — Additional file 6: Figure S5. Scatter diagrams for module membership vs. gene significance of disease state in brown(A), magenta(B), red(C) and grey(D) module. [file 12967_2020_2463_MOESM6_ESM.tif]

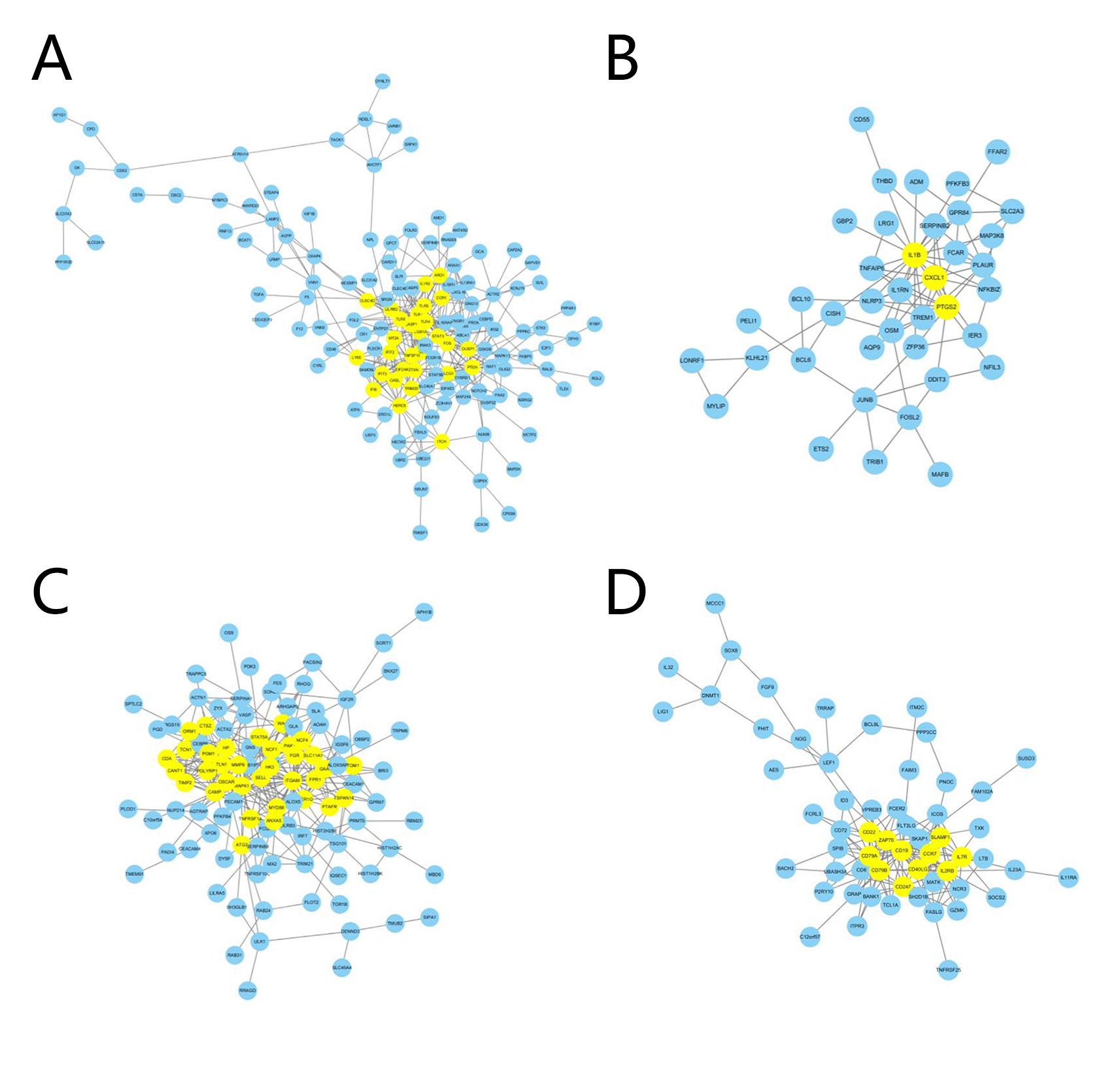

Supplement: Supplementary file 7 — Additional file 7: Figure S4. PPI network in different modules. (A) Yellow module. (B) Black module. (C) Blue module. (D) Turquoise module. The yellow nodes represent the hub genes (connectivity degree > 8) in each module. [file 12967_2020_2463_MOESM7_ESM.tif]

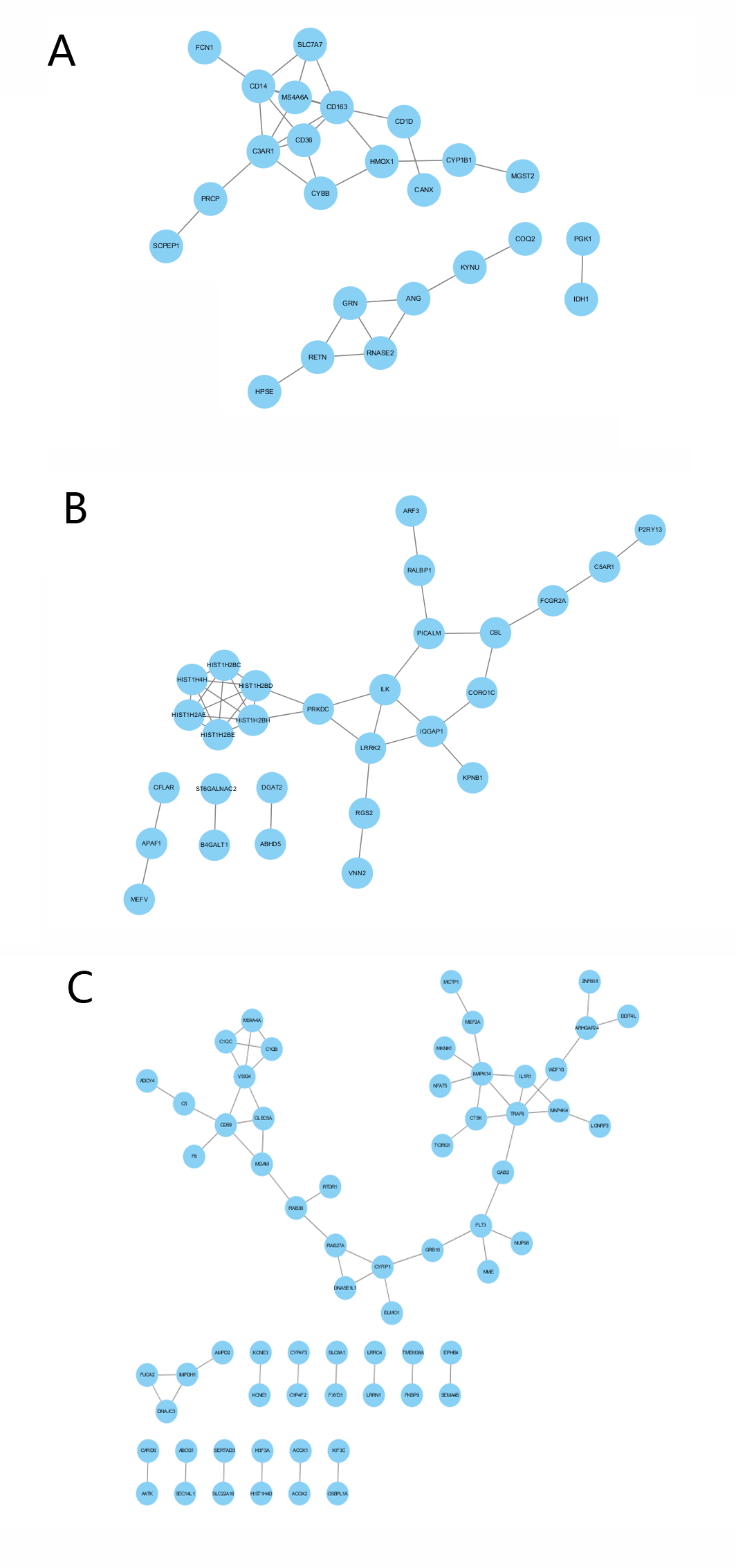

Supplement: Supplementary file 8 — Additional file 8: Figure S6. PPI networks of purple(A), pink(B) and green(C) module. [file 12967_2020_2463_MOESM8_ESM.tif]
