# Supplementary material for: Identifying the pattern of immune related cells and genes in the peripheral blood of ischemic stroke
Source: J Transl Med. 2020 Aug 3;18:296. doi: 10.1186/s12967-020-02463-0 (PMC7398186; doi:10.1186/s12967-020-02463-0)
Supplement: Supplementary file 2 — Additional file 2: Table S1. Sequences of primers used for qPCR. Table S2. Clinical characteristics of validation samples. [file 12967_2020_2463_MOESM2_ESM.docx]

**Table S1. Sequences of primers used for qPCR.**

| Gene | Gene bank accession |  | Sequence |
| --- | --- | --- | --- |
| IL7R | NM_002185 | forward | 5’-AGAAAGTGGCTATGCTCA-3’ |
|  |  | reverse | 5’-ACATCTGGGTCCTCAAAA-3’ |
| ITGAM | NM_000632 | forward | 5’-GGCGGATGAAGGAGTTTG-3’ |
|  |  | reverse | 5’-TCTTGGGTTAGGGTTGTT-3’ |
| NCF4 | NM_000631 | forward | 5’-TTTTACCAGTCGCCCTAT-3’ |
|  |  | reverse | 5’-CTCCAGTTTGCTGTTTCC-3’ |
| PAK1 | NM_002576 | forward | 5’-CCAAACCCAGAGGAGAAG-3’ |
|  |  | reverse | 5’-ACAGCATCAAAACCGACA-3’ |
| PTEN | NM_000314 | forward | 5’-GCCACAGGCTCCCAGACA-3’ |
|  |  | reverse | 5’-TTTACCCAAAAGTGAAACATT-3’ |
| MYD88 | NM_002468 | forward | 5’-CTGGAACAGACAAACTATCG-3’ |
|  |  | reverse | 5’-AGACAACCACCACCATCC-3’ |
| FGR | NM_005248 | forward | 5’-AAGCATTACAAGATCCGCAAAC-3’ |
|  |  | reverse | 5’-CACAGCCCGTCATTCACCT-3’ |
| SLAMF1 | NM_003037 | forward | 5’-GGAAAGCAGGAAGGAGGA-3’ |
|  |  | reverse | 5’-GCAGCCCAGTATCAAGGT-3’ |
| TLR8 | NM_016610 | forward | 5’-TCTTCTCGGCCACCTCCT-3’ |
|  |  | reverse | 5’-ATGGGTCTTGCTGATGTTCC-3’ |
| ATG7 | NM_006395 | forward | 5’-TGGAACAAGCAGCAAATG-3’ |
|  |  | reverse | 5’-ACTGGCCCCTGAATGAGA-3’ |
| MAPK1 | NM_002745 | forward | 5’-AGGCTGTTCCCAAATGCT-3’ |
|  |  | reverse | 5’-CTCGTCACTCGGGTCGTA-3’ |
| IFNAR1 | NM_000629 | forward | 5’-CATGGATGAAAAGCTGAA-3’ |
|  |  | reverse | 5’-TAAATGACAAACGGGAGA-3’ |
| CCR7 | NM_001838 | forward | 5’-AGACAGGGGTAGTGCGAGG-3’ |
|  |  | reverse | 5’-ACCACCAGCACGCTTTTC-3’ |
| TIMP2 | NM_003255 | forward | 5’-CTGCTGGCGACGCTGCTT-3’ |
|  |  | reverse | 5’-TTCTCACTGACCGCTTTGGC-3’ |
| CANT1 | NM_138793 | forward | 5’-TGCCCTGGGTGATTCTGT-3’ |
|  |  | reverse | 5’-TGGTCGTCCACTCCTTGC-3’ |
| WAS | NM_000377 | forward | 5’-TTGAGATGCTTGGACGAAA-3’ |
|  |  | reverse | 5’-AAAGGCGGATGAAGTAGGA-3’ |
| GAPDH | NM_002046 | forward | 5’-AGGGCTGCTTTTAACTCTG-3’ |
|  |  | reverse | 5’-CTGGAAGATGGTGATGGG-3’ |

**Table S2. Clinical characteristics of validation samples.**

|  | IS patient group (n=15) | | Control group (n=15) | |
| --- | --- | --- | --- | --- |
| Sex | Male | Female | Male | Female |
| Numbers | 8 | 7 | 5 | 10 |
| The median age (year-old) | 60(34-75) | 59(55-80) | 59(36-73) | 69.5(55-84) |
